# Health care for irregular migrants: pragmatism across Europe. A qualitative study

**DOI:** 10.1186/1756-0500-5-99

**Published:** 2012-02-16

**Authors:** Marie Dauvrin, Vincent Lorant, Sima Sandhu, Walter Devillé, Hamidou Dia, Sónia Dias, Andrea Gaddini, Elisabeth Ioannidis, Natasja K Jensen, Ulrike Kluge, Ritva Mertaniemi, Rosa Puigpinós i Riera, Attila Sárváry, Christa Straßmayr, Mindaugas Stankunas, Joaquim JF Soares, Marta Welbel, Stefan Priebe

**Affiliations:** 1 Fonds de la Recherche Scientifique-FNRS, rue d'Egmont 5, 1000 Bruxelles, Belgium; 2 Institute of Health and Society IRSS, Université catholique de Louvain, Clos Chapelle aux Champs 30 B1.30.15, 1200 Bruxelles, Belgium; 3 Unit for Social and Community Psychiatry, London and the Barts School of Medicine and Dentistry, Queen Mary University of London, Newham Centre for Mental Health, London E13 8SP, UK; 4 International and Migrant Health, NIVEL (Netherlands Institute for Health Services Research), Otterstraat 118-124, PO Box 1568, 3500, BN Utrecht, The Netherlands; 5 Etablissement public de santé Maison Blanche, 3-5 rue Lespagnol, 75020 Paris, France; 6 Instituto de Higiene e Medicina Tropical, Universidade Nova de Lisboa, Rua da Junqueira, 96, 1349-008, Lisbon, Portugal; 7 Laziosanità ASP Public Health Agency for the Lazio Region, Via S. Costanza 53, 00185 Rome, Italy; 8 Department of Sociology, National school of Public Health, 196 Alexandras avenue, Athens 11521, Greece; 9 Danish Research Centre for Migration, Ethnicity and Health (MESU), Section for Health Services Research, Department of Public Health, University of Copenhagen, Øster Farimagsgade 5, DK-1014 Copenhagen, Denmark; 10 Clinic for Psychiatry and Psychotherapy, Charité - University Medicine Berlin, CCM, Charitéplatz 1, 10117 Berlin, Germany; 11 Department for mental health and substance abuse services, National Institute for Health and Welfare (THL), P.O.B. 30, FIN-00271 Helsinki, Finland; 12 Agency of Public Health of Barcelona, Pça. Lesseps, 1, 08023 Barcelona, Spain; 13 Faculty of Health, University of Debrecen, Sóstói út 2-4, 4400 Nyíregyháza, Hungary; 14 Ludwig Boltzmann Institute for Social Psychiatry, Lazarettgasse 14A-912, 1090 Vienna, Austria; 15 School of Public Health, Griffith University, Gold Coast Campus, Southport, Queensland 4222, Australia; 16 Department of Health Management, Lithuanian University of Health Sciences, A. Mickeviciaus 9, Kaunas 44307, Lithuania; 17 Department of Public Health Sciences, Section of Social Medicine, Karolinska Institutet, SE- 171 76 Stockholm, Sweden; 18 Department of Public Health Sciences, Mid Sweden University, SE-851 70 Sundsvall, Sweden; 19 Institute of Psychiatry and Neurology, Ul. Sobieskiego 9, 02-957 Warsaw, Poland

**Keywords:** Irregular migrants, Europe, Qualitative method, Health services, Accessibility

## Abstract

**Background:**

Health services in Europe face the challenge of delivering care to a heterogeneous group of irregular migrants (IM). There is little empirical evidence on how health professionals cope with this challenge. This study explores the experiences of health professionals providing care to IM in three types of health care service across 16 European countries.

**Results:**

Semi-structured interviews were conducted with health professionals in 144 primary care services, 48 mental health services, and 48 Accident & Emergency departments (total n = 240). Although legal health care entitlement for IM varies across countries, health professionals reported facing similar issues when caring for IM. These issues include access problems, limited communication, and associated legal complications. Differences in the experiences with IM across the three types of services were also explored. Respondents from Accident & Emergency departments reported less of a difference between the care for IM patients and patients in a regular situation than did respondents from primary care and mental health services. Primary care services and mental health services were more concerned with language barriers than Accident & Emergency departments. Notifying the authorities was an uncommon practice, even in countries where health professionals are required to do this.

**Conclusions:**

The needs of IM patients and the values of the staff appear to be as important as the national legal framework, with staff in different European countries adopting a similar pragmatic approach to delivering health care to IM. While legislation might help to improve health care for IM, more appropriate organisation and local flexibility are equally important, especially for improving access and care pathways.

## Background

Irregular migrants (IM) are considered to be non-nationals without legal authorisation to stay in Europe on regular terms [1,2]. In 2008 the estimates for the 27 European Union (EU) states were that 1.9 to 3.8 million people were IM, with patterns of distribution varying between countries [2]. Countries such as Germany or France have extensive experience of IM, while relatively new EU member states, such as Lithuania or Hungary, have only recently become hosts to IM. Public authorities and health agencies have to cope with different groups of IM that experience various difficulties in accessing health care or meeting their specific health needs [3-6]. IM are vulnerable to infectious diseases, psychiatric disorders, and digestive and osteoarticular problems, as well as emotional distress and poor subjective health [7-11].

The obstacles faced by IM when attempting to access and use health care services have been identified from the patient perspective [1,7,8,11] or in legislation [12-15]. However, research has rarely approached the issue from the perspective of health professionals: what the challenges encountered by professionals are when delivering health care to IM across Europe and how they attempt to meet them.

International differences in legal access to care for IM may lead to different practices. Several classifications have attempted to clarify the complex situation of access to care for IM [1,10,15]. The *NowHereland *project identified three categories of health care entitlement for IM in the EU: "no rights", "minimum rights", and "rights" [16,17]. The "no rights" category applies when health care access is restricted to such an extent that emergency care is inaccessible. The "minimum rights" category applies where IM can access emergency care or similar services. The "rights" category applies where IM can access services other than emergency care, such as primary and secondary care.

To explore how services approach access and delivery of health care to IM, as experienced by health professionals across Europe, we carried out some research as part of the EUGATE project. The EUfinanced EUGATE project aims to assess "Best Practice in Health Services for Immigrants in Europe" in sixteen countries: Austria, Belgium, Denmark, Finland, France, Italy, Lithuania, Germany, Greece, Hungary, Netherlands, Poland, Portugal, Spain, Sweden, and the United Kingdom [18-20]. EUGATE targeted five groups of migrants: labour migrants, refugees, asylum seekers, victims of human trafficking, and IM.

In this paper, we aim to identify the specific challenges experienced by professionals in clinical encounters with IM, with particular attention being paid to the differences between types of services and countries involved in EUGATE. Although there were already several publications from EUGATE, we decided to focus on the particular situation of IM, a particularly vulnerable group for which the European Council has called for more attention. We expected differences between countries and between services due to different legal entitlements. Focusing on IM also allowed us to assess whether legal status has an impact on health professionals' attitudes.

## Results

Of the total sample, 83.5% of the interviewees were clinicians (mostly doctors and nurses) and 16.5% worked as managers within the institutions.

After completing the interviews, we identified three main themes relating to IM: access, notifying the authorities, and communication. We then compared services and countries. Countries were grouped according to the NowHereland categories: "no rights", "minimum rights, and "rights" [16,17] [see Table 1 for the classification of countries]. NowHereland categories were chosen as possible explanations for differences between countries.

**Table 1:** Classification of European countries according to healthcare entitlement for irregular migrants a, b

No Rights	Minimum Rights	Rights
Finland	Austria	France
Sweden	Belgium	Italy
	Denmark	Netherlands
	Germany	Portugal
	Greece	Spain
	Hungary	
	Lithuania	
	Poland	
	United Kingdom	

^a ^Categories based on the typology developed in the *NowHereland *project [16,17]

^b ^We present only the countries involved in the EUGATE project.

### Access

Because of differences in the organisation and funding of health care systems across participating EUGATE countries, "access" could mean either entitlement or affordable care [21].

All professionals in A&E departments, even in countries belonging to the "no rights" category, reported full access to IM (n = 48). However, for primary care, respondents in 16 services (n = 144) reported that no access was provided, either because of the absence of legal entitlement or due to financial barriers. Interviewees reported that treatment in A&E departments may be provided in emergency situations without clarification of the patient's entitlement to treatment being required. Furthermore, most A&E professionals reported no differences in delivering health care for IM and patients in a regular situation.

In mental health services, a quarter of respondents reported that it was unlikely for IM to come to their services for treatment. But they stated they would, nevertheless, provide care for an IM should they access the service.

Respondents in 24 A&E departments declared that they provide the same further treatment pathways for IM as for patients in a regular situation. Professionals in primary care and mental health services experienced more difficulties in performing further diagnostic and/or therapeutic interventions due to the restricted access IM face in health care. Prescribing drugs could be really difficult, as the IM patient could not afford it.

*...because he is not health insured I will not give him a prescription - I cannot give him a prescription and I don't want to give him a private prescription, because he cannot afford it. That's why I actually always solve that problem by giving free medical samples and that works wonderfully..*.

ID7, primary care services, Austria

*...I prescribe the medicines for my own name, if the patient has no money for it..*.

ID 146, primary care services, Hungary.

Difficulties in continuity of care occurred when supplementary treatment was arranged within the same service or when IM had to be referred to another service. This situation was reported even in countries where IM were guaranteed full rights.

*If I have to refer him, it will be a hassle (I'm not saying it's not possible). So you can assume that a doctor will have a certain threshold for consulting a specialist. This is also to avoid getting the patient into trouble. For example, if I refer him to the emergency department, he could subsequently receive an 800 euro bill when there's nothing really wrong with him*.

ID 206, primary care services, Netherlands

*Where it gets complicated is if they need a referral to the hospital. That's where it gets complicated because, although we always do our bit, when they get to the hospital end, they may be charged. The situation at the hospital will be very different, because very different criteria are involved. We don't get involved in these situations. They may or may not be seen by a specialist, if that is what they need. It's not something we can control*.

ID 312, primary care services, UK

Some professionals, especially in primary care, reported transferring IM between services or having to delay treatment while waiting for legal issues surrounding the patient's irregular status to be resolved.

*An illegal patient would be received and treated illegally...such a patient is entitled to basic medical treatment only. And in theory - they should cover the treatment costs. If they had money, they could pay for the visit and receive a full range of services. And if not, emergency care and basic treatment only.... And perhaps, a doctor would arrange a check-up visit for them, without registering it..*.

ID 234, primary care services. Poland

Interviewees in primary care and mental health services also reported informing patients about services that provide free health care and administrative support or referring them directly to such services in order to bypass problems with access in their own service.

### Notifying the authorities

Primary care and A&E departments were compared in terms of notifying public authorities, including the police, about IM being treated in their service. Most interviewees in both services reported that they would not inform the police about IM presenting at their service for treatment. Few would inform the police: 10 in primary care services (n = 144) and 5 in A&E departments (n = 48). In both types of service the tendency to inform the police was stronger in cases where the patient was suspected of being involved in criminal activities.

Conversely, some respondents reported informing the police to help IM or to protect them. Such scenarios included situations in which patients were considered a danger to themselves or to others. The need to identify the patient in critical situations would qualify as reason enough to inform the authorities.

*No. For several reasons. There is no need for me to inform the police. If the individual was severely injured and he got so bad that you would need to get in touch with relatives or the like you would try through the police. Normally, we will contact the police if we are to get hold a relative we do not know about. If we have an actual identification problem and a need to inform relatives. That situation can arise if it is a catastrophic situation, but otherwise there is no need to contact the police*.

ID40, A&E department, Denmark

*By hook or by crook, we would find out who he is. Insurance, marital status, police, foreigners department.... No idea, we cannot take him into custody to move him into prison hospital*.

ID 100, A&E department, Germany

Finally, although many of the interviewees would not inform the police, six primary care services and one A&E department did inform their own managerial staff. This was usually due to financial and organisational problems for which management would be responsible.

### Communication

In primary care and mental health services, communication barriers were perceived as more problematic than in A&E departments. In A&E departments, staff emphasised difficulties in reaching a diagnosis due to language barriers, while professionals in primary care and mental health services reported communication difficulties being a more general problem. Interviewees from primary care services discussed issues associated with patients becoming more stressed as a result of not being able to express themselves to professionals.

Health professionals recommended or used professional face-to-face interpreters or telephone interpreting services when they had language barriers with their patients. However staff still reported little use of these interpreting services or not having full access to them. This was attributed to the administrative procedures involved, the lack of funding, or the poor quality of interpreting when available. Consequently, in practice, professionals, especially in primary care services, arranged alternative solutions such as asking children, families, friends, or bilingual employees to act as interpreters.

*[...]So that's my Chinese I've told you about, an illegal immigrant. She was working illegally in a Chinese restaurant and I found another Chinese restaurant, and what happened is that they were able to communicate and the owner of that Chinese restaurant who was used to talk with her explained to her that she had appendicitis and that she had to be operated on. And when I told him "one should tell her that", he said "Oh appendicitis, I must look that up in a dictionary" not for the translation but to know what it was, he had never heard that word in French before so he looked the word up and told her she had to be operated on..*.

ID 32, Accident & Emergency Department, Belgium

Among non-major themes some interviewees reported problems related to culture, such as the refusal of care due to the health professional being of the opposite gender or due to cultural beliefs that hindered recovery. Two health professionals out of the whole sample reported different expectations about treatment leading to misunderstanding between health professionals and IM.

### International comparisons

Despite variations in health care entitlement for IM, most of the countries investigated faced similar issues. No important differences in frequency were noted between countries in the "rights" category and those with only "minimum rights" for IM. In countries in the "no rights" category, communication problems and their consequences were the main theme. "Access problems and their consequences" were cited in all Swedish services while in Finnish services this theme was reported in only 2 services (n = 15).

Differences were found between countries regarding notifying the police about IM. In 5 of the 16 countries, interviewees considered informing the police about an IM treated in the service. Three of these countries are considered to provide "minimum rights", while the remaining two were classified as "no rights" countries [Table 1]. Although they were classified in the "minimum rights" category, Germany and Lithuania were, during the data collection period, the two countries where there was an obligation of notifying IM to the police. However, only a few health professionals from both countries reported having done this.

Although we expected differences due to different legislation regarding access to health care, a quarter of the interviewees stated that there were no differences in the actual care provided for IM compared with patients in a regular situation in the host country.

Nevertheless, although in some countries IM were legally entitled to a wide range of health care services, professionals reported insufficiencies in the actual delivery of care. Where patients did have access to services, the quality of care was reported to be poor due to lack of funds, administrative requirements, and practices and procedures within the service. Consequently, some professionals reported transferring IM to other health care services with better human or material resources. Some professionals suggested that they would consider transferring patients, even when they were allowed to care for them or had the required funds to do so, to avoid the burden of IM on the service. Non-governmental organisations (NGOs) caring for IM were quoted as potential referral agencies, particularly in countries where IM do have access to care, such as Belgium or France.

## Discussion

We aimed to identify the challenges experienced by professionals in clinical encounters with IM, with a view to highlighting differences between services and countries. Comparisons between services and countries centred on three themes retrieved from interviews with practitioners and managers: access, notifying the authorities, and communication. Language barriers and restricted access to adequate treatment in the service and further treatment pathways limit the therapeutic options available, lower the quality of care, and jeopardise the continuity of care [22]. These themes are consistent with previous studies among IM or migrants in a regular situation and form a relevant basis for comparison [23,24].

Staff in primary care and mental health services mainly reported problems relating to language barriers. Although they reported full access to their service, staff in A&E departments mainly reported access issues. In fact, accessing the service does not prevent patients from having other access problems. IM may have to pay extra costs that may prevent them from using the service subsequently. Irregular status may restrict the care available or further treatment. Access had several meanings for the interviewees: in addition to entitlement to care, these included geographical accessibility and financial accessibility. These differences between services may also be linked to the different tasks they perform and the different treatments they provide. Emphasis on talk-based assessment and treatment in mental health requires clear communication for effective outcomes. Further research is needed to explore the influence of clinical tasks and treatment pathways on the problems experienced by health professionals when caring for IM. In addition, most EU countries have gatekeeping systems for specialised health services, as mental health care [25]. Thus, referral by health professionals may often be required to access mental health care but not A&E departments. This may also explain why professionals in mental health services declared that having an IM patient was rather unlikely for them. Out-of-pocket payments, which may be significant in some countries, could also prevent IM from accessing mental health services.

Although some themes were more commonly reported in some groups of countries than in others, no important differences were observed between countries. The most frequent themes were reported across all of the *NowHereland *country categories: "no rights", "minimum rights", and "rights". Nor did self-reported professional practice differ greatly between countries.

There are four possible reasons for the relatively few differences between countries. Firstly, in countries with "minimum rights" (and assuming the same for "no rights" countries), health professionals may treat IM even though they have no or limited entitlement to access the service. Some professionals considered treating the patient to be more important than abiding by the law [19]. Staffs tend to adopt a more pragmatic approach, which is reflected by their willingness to provide care even where legislation restricts access to health care for IM [26,27].

Alternatively, they may also apply institutional guidelines or policies regarding health care for IM. This may be the result of broader health policies aimed at improving access to care and quality of care for migrants. Examples of such policies are the "Checking for Change" programme developed by NHS Scotland or the "Migrant-Friendly Hospitals" network. Policies of this kind may increase health professionals' awareness of ethnicity and migration issues. As health professionals may become more sensitive to migrants' needs, this could positively influence them when caring for IM.

Consequently, values other than laws may be the drivers when delivering health care to IM. In addition, health professionals sensitive to migration issues tend to work in places where most patients are migrants [28]. These professionals may have a greater familiarity with and empathy for IM issues, and therefore be more inclined to facilitate access to care.

Secondly, there are divergent interpretations of concepts such as "basic health care", "right to health", and "health care accessibility" within and between countries, services, and professionals. For example, in Lithuania, one interviewee reported equal access to primary care services, while a second reported no access and a third reported restricted access. Based on our findings, it seems that divergent interpretations of legislation may improve access to care at the national level. This is obviously a contentious topic. Indeed, previous studies have reported divergent interpretations of legislation as a barrier to health care for IM [8,14]. From the IM's perspective, divergent interpretations introduce an element of unpredictability in terms of the delivery of health care: IM do not know what their situation is until they see the doctor. The effects of such uncertainty on access deserve more research.

Thirdly, a lack of awareness of the legal requirements for delivering care to IM could be common among health care staff. Indeed, previous studies have shown that staff are not always aware of the legal framework as it applies to health care delivery [29,30]. In the absence of an incentive framework to deliver health care for IM, professionals' decision-making may be guided above all by their professional values [26,31]. They may, as a consequence, deliver health care according to patient need. Therefore implicit rationing may also play a role, especially in a context of scare resources [32]. Such situations may also be unfavourable to IM. It was not uncommon for health professionals in "rights" countries to refer IM to other health services such as NGOs or charitable institutions, despite favourable laws entitling IM to health care.

Finally, even if professionals are aware of the laws regulating health care for IM patients, the procedures for implement these may be inadequate. In health care, putting legal or health policies into practice at the service level may be problematic and may be subject to different interpretations [33,34]. Consequently, applying the legislation may be left to the discretion of the staff within the service, leading to decisions being made on a case-by-case basis [35,36]. However, even if health professionals tend to bend the rules, IM may not benefit from such flexible attitudes as they may not be aware of them. Moreover, uncertainty may increase distrust of health care providers in general. Information about "flexible health professionals" will spread unequally across informal networks of assistance and may fail to reach very isolated IM.

No differences in communication issues were observed between countries. All migrants potentially face language barriers when accessing health care systems and these barriers may persist for years after their arrival [37,38].

This study had certain limitations. It is possible that, especially considering the convenience sampling method used, respondents may have given socially desirable answers, leading to underestimation of problems such as racism or restricted access.

The actual practice in services was not assessed; interviewees reported their own subjective experiences. Staff were interviewed about potential patients who had already presented, having somehow accessed the service, although their entitlement to receive appropriate care may have been restricted. However, even in countries where IM were not fully entitled to access services, staff nevertheless reported that they tried to be helpful and found ways of providing care for IM. Further studies should triangulate results with IM medical records to help assess the effective restriction of care. Further research could be conducted in cooperation with NGOs with extensive experience in registering IM patients' data, such as Doctors of the World. Alternative research methods, such as the snowball approach and other social network methods, may help us in improving some aspects of triangulation [39].

We selected areas in large cities with high levels of immigration but the situation in rural areas with lower numbers of migrants may be significantly different. Nor do we know how representative the interviews are for other services in the same areas.

Finally, our sample of European countries includes far fewer "no-rights" (n = 2) countries than countries classified as providing "rights" or "minimum rights" (n = 14), when compared with the *NowHereland *study (9 and 18, respectively). Our results may therefore present a somewhat optimistic picture of the situation in the EU. Further studies may involve all EU countries. Moreover, as the entitlement to health care for IM is still evolving in Europe, new practices may emerge and modify our findings.

## Conclusions

This study was one of the first to use a similar methodology across 16 countries to explore practices for treating IM in three types of services. Its aim was to fill the gap in research between macro-level dispositions ensuring access to health care for IM, and staff's daily practice. It is crucial that entitlements to health care as defined by laws and policies be analysed separately from actual access to health care services on the ground [15]. Differences between legislation and practice have been previously reported from the perspective of IM, but not by health professionals [8]. Such differences may also help with designing solutions designed to ultimately improve access to care for IM.

Access to health care seems to be grounded on the basis of the needs of the IM rather than on their legal status in regards to health care. Some staff reported seeking to care for patients on a case-by-case basis and based on their legal entitlement to care. For professionals, one possible common value is to provide health care regardless of legal or political restrictions. A "deep rooting" of humanism in the minds of health professionals has been reported as a crucial determinant of access to health care for IM [12]. In practice, more factors than legislation alone determine the access to health care of IM [12,15].

Although most practice was based on pragmatic decisions about delivering health care for IM as efficiently and equitably as possible, clear legislation to ensure access to care for IM was also necessary. It is clear that the quality of care for IM patients could be improved by enacting legislation where it is needed [18,19,40]. In the absence of uniform legislation that ensures funding for all types of health care that IM could require, there should at least be clarification of the existing funding rules and local flexibility in terms of arranging health care for IM. Countries with "minimum rights" to health care must maintain their efforts to increase access to outpatient services, notably by reducing the fees and the administrative procedures involved. Finally, more vulnerable groups such as children, the elderly and pregnant women require special attention [15].

Modifying legislation is an important step for countries classified in the "no rights" or "minimum rights" categories, but this alone may not be enough to raise the quality of care. One may conclude from this study that there are ways to be a "pragmatic health professional" when providing health care for IM in different services by working with the available resources and seeking alternative routes of access where possible. NGOs and other charitable organisations, for example, remain an important support to mainstream health institutions in terms of providing health care to IM. This appears to apply to all countries, even those with different "rights" approaches. Taking a pragmatic approach when delivering care to IM, along with improvements to organisational and local flexibility at both policy and practice level, may contribute to improving both the quality of care delivered and the pathways into care.

### Availability of supporting data

The full questionnaire of the EUGATE project is available in an Additional file 1.

## Method

### Setting

In each country, three administrative districts, each with a high proportion of migrants (based on available data or informed estimates), were selected, mostly within the country's capital. Three different services were sampled per zone: Accident and Emergency departments (A&E), primary care services, and mental health services. From those services with the highest percentages of migrant patients, the services with the greatest numbers of patients were selected. The respondents were personnel working within these services who had knowledge and practical experience of providing health care to migrants [18,19]. Within each area, all identified services were contacted directly and no sampling selection was required. Participation was on a voluntary basis. Although there was no systematic registration of refusals, refusal was rather rare. As interviewees were chosen because they were believed to be providing care for migrants, it is likely that very few health professionals refused to participate when migrants were an important target group.

Semi-structured interviews were conducted with health professionals in 144 primary care services, 48 mental health services, and 48 A&E departments, across 16 European countries (total n = 240). In each country, the sample was made up of 3 mental health services, 3 A&E departments, and 9 primary care services.

Informed consent was obtained prior to the interviews, and the study was approved by the relevant ethics committees in countries where this was required. Ethics approval for the study was obtained in Portugal through the University Hospital S. João. In other countries ethics approval was not required because no patient data was recorded, and the study was regarded as service evaluation without need for ethical review under the conditions set out in the Helsinki Declaration for research conducted with human participants.

### Data collection

Semi-structured interviews were conducted in 2008-2010. The interview schedule was developed in English, piloted in each country, and translated into local languages. The first part of the interview was made up of questions about general experiences when providing health care to migrants. These questions about general experience were open questions about 1) the specific problems encountered with all migrant patients, 2) good practice when delivering services to immigrants, and 3) the need to improve the services' care for such target groups [Table 2]. The second part of the interview consisted of vignettes about IM. The case vignettes allowed the health professionals to describe their practical experiences with IM [41].

**Table 2:** Clinical vignettes submitted to health professionals

	Primary care Services	A&E department	Mental health Services
**Clinical Situation**	A male, 28 years old, coming from [*insert a country*], presents with pain when urinating and has a slight fever. He does not speak any language that the doctor understands. He has no insurance, no identification, and no residency permit.	The patient arrived in the host country as an irregular immigrant about 1 year ago. He is 25 years of age and of [*insert a country*] origin. He does not speak any language that the A&E staff understands and presents with an intense lower abdominal pain.	The patient arrived in the host country as an irregular immigrant about 1 year ago. She is 25 years of age and of [*insert a country*] origin. She does not speak the language of the host country, has no social contacts and appears severely depressed.

**Question 1**	From your perspective, what are the differences, if any, in the treatment for this patient compared to a patient with a similar condition from the indigenous population?		

**Question 2**	From the perspective of a patient, what do you think are the specific problems this patient would encounter that are different from those of a patient with a similar condition from the indigenous population, and how would they be overcome?		

**Question 3**	What are the specific further pathways and treatment options, if any, for this patient that are different from those of a patient with a similar condition from the indigenous population?		

**Question 4a**†	Would you inform the police and/or other authorities?		

**Question 4b**‡	Is this scenario at all possible, or are there barriers preventing irregular immigrants from using your service?		

† For respondents in primary care services and A&E departments only.

‡ For respondents in mental health services only. Due to the gatekeeping systems that exist in most European countries for mental health services, even for legal patients, the likelihood of an IM presenting in mental health services was investigated [25]. Mental health services were selected as examples of secondary services.

The case vignettes focused on the management of clinical situations involving IM patients as compared to situations involving patients in a regular situation in the given host country. Specific questions about informing the authorities or the police were submitted to A&E departments and primary care services. One specific question was asked of mental health services only: whether or not IM would ever receive treatment from their service. Interviews (238/240) were taped. Two participants out of the whole sample refused to have their interviews recorded; their responses were documented in writing. All interviews (240) were transcribed verbatim.

### Analysis

A thematic content analysis was conducted based on interview responses [42]. Each centre first generated a list of emerging codes in a stepwise analysis, based on a line-by-line analysis of the first three interviews carried out in the country in which it was located [43]. National research centres created an inductive code list based on the material.

The UK coordinators received a database containing the codes and the associated text translated into English for each country. The codebook was developed based on the data provided.

Consistency of coding was assessed across all participating centres. Firstly, researchers at the coordinating centre screened the databases containing the coding results of the first interviews in each centre and discussed any discrepancies with the relevant centre. Secondly, researchers from all centres provided further verification and clarification of the meanings of the codes during a one-day workshop. Thirdly, each partner coded two interviews conducted in English and the UK coordinating centre assessed the coded data for discrepancies. The themes were organised according to the current scientific literature exploring access to and delivery of care for migrants.

The smallest textual unit of analysis was a single sentence. Centres reviewed and agreed upon a codebook based on the data. Interviews were coded with the codebook. Codes were categorised based on their English translations. To obtain meaningful themes, the emerging categories and codes were organised and grouped [41,44,45]. Researchers from all centres verified the emerging categories and themes to ensure consistency across the data set. Descriptive counts of themes, categories, and codes summarised the data set.

Further details of the design and methods of analysis are reported elsewhere [18,19].

## Abbreviations

IM: Irregular Migrants; EU: European Union; A&E department: Accident and Emergency department; UK: United Kingdom; NGOs: Non-Governmental Organisations

## Competing interests

The authors declare that they have no competing interests.

## Authors' contributions

MD supported study coordination for the Belgian part of the study, analysed the data, and drafted the manuscript. VL was in charge of the Belgian part of the study, participated in data analysis, and helped draft the manuscript. SP designed the study protocol, was in charge of the overall management of the study, and helped draft the manuscript. SS supported study co-ordination. All authors collected national data and are guarantors of the data for the countries they dealt with; they commented on successive drafts of the manuscript and approved its final version.

## Supplementary Material

Additional file 1 **European Best Practices in Access, Quality and Appropriateness of Health Services for Immigrants in Europe**. Click here for file

## References

[B1] PICUM Access to Health Care for Undocumented Migrants in Europe 2007 Brussels: PICUM 10.1016/S0140-6736(07)61872-8 18156010

[B2] CLANDESTINO group Undocumented Migration: Counting the Uncountable. Data and Trends Across Europe. Final Report 2009 Brussels: European Commission

[B3] Castañeda H Illegality as risk factor: A survey of unauthorized migrant patients in a Berlin clinic Soc Sci Med 2009 68 8 1552 1560 10.1016/j.socscimed.2009.01.024 19246143

[B4] Dorn T Ceelen M Tang MJ Browne JL de Keijzer KJC Buster MCA Das K Health care seeking among detained undocumented migrants: a cross-sectional study BMC Public Health 2011 11 190 10.1186/1471-2458-11-190 21443761 PMC3078097

[B5] Florence S Lebas J Parizot I Sissoko D Querre M Paquet C Lesieur S Chauvin P Migration, health and access to care in Mayotte Island in 2007: Lessons learned from a representative survey Rev Epidémiol Santé Publique 2010 58 4 237 244 10.1016/j.respe.2010.04.006 20634011

[B6] Wolff H Epiney M Lourenco A Costanza MC Delieutraz-Marchand J Andreoli N Dubuisson J-B Gaspoz J-M Irion O Undocumented migrants lack access to pregnancy care and prevention BMC Public Health 2008 8 93 10.1186/1471-2458-8-93 18366690 PMC2323378

[B7] Cavazos-Rehg PA Zayas LH Spitznagel EL Legal status, emotional well-being and subjective health status of Latino immigrants JAMA 2007 99 10 1126 1131 PMC2574408 17987916

[B8] Chauvin P Parizot I Drouot N Simonnot N Tomasino A European Survey on undocumented migrants' access to health care 2007 Paris: European Observatory of Doctors of the World21944556

[B9] Chauvin P Parizot I Simonnot N Access to health care for undocumented migrants in 11 European countries 2009 Paris: European Observatory of Doctors of the World

[B10] Sebo P Jackson Y Haller Dagmar DM Gaspoz J-M Wolff H Sexual and Reproductive Health Behaviors of Undocumented Migrants in Geneva: A Cross Sectional Study J Immigr Minor Health 2011 13 3 510 517 10.1007/s10903-010-9367-z 20623192

[B11] Pikhart H Drbohlav D Dzurova D The self-reported health of legal and illegal/irregular immigrants in the Czech Republic Int J Public Health 2010 55 5 401 411 10.1007/s00038-010-0156-1 20552249

[B12] Romero-Ortuño R Access to health care for illegal immigrants in the EU: should we be concerned? Eur J Health Law 2004 11 245 272 10.1163/1571809042388572

[B13] HUMA network Law and practice: Access to health care for undocumented migrants and asylum seekers in 10 EU countries 2009 Paris: HUMA network

[B14] Torres-Cantero AM Miguel AG Gallardo C Ippolito S Health care provision for illegal migrants: may health policy make a difference? Eur J Public Health 2007 17 5 483 485 17208953 10.1093/eurpub/ckl266

[B15] Ruiz-Casares M Rousseau C Derluyn I Watters C Crepeau F Right and access to healthcare for undocumented children: Addressing the gap between international conventions and disparate implementations in North America and Europe Soc Sci Med 2010 70 2 329 336 10.1016/j.socscimed.2009.10.013 19892452

[B16] Björgren Cuadra C Cattacin S NOWHERELAND Policies on Health Care for Undocumented Migrants in the EU27: Towards a Comparative Framework 2010 Malmö: NOWHERELAND

[B17] Björgren Cuadra C Right of access to health care for undocumented migrants in EU: a comparative study of national policies Eur J Public Health 2011 first published online June 9, 2011 doi:10.1093/eurpub/ckr049 10.1093/eurpub/ckr049 21659389

[B18] Priebe S Bogic M Adány R Bjerre NV Dauvrin M Devillé W Dias S Gaddini A Greacen T Kluge U Ioannidis E Jensen NK Puigpinós I Riera R Soares JJF Stankaunas M Straßmayr C Wahlbeck K Welbel M McCabe R Rechel B, Mladovsky P, Devillé W, Rijks B, Petrova-Benedict R, McKee M Good practice in emergency care: views from practitioners Migration and Health in the European Union 2011 First Maidenhead: Open University Press 213 226

[B19] Priebe S Sandhu S Dias S Gaddini A Greacen T Ioannidis E Kluge U Krasnik A Lamkaddem M Lorant V Puigpinós i Riera R Sárváry A Soares JFF Stankunas M Straßmayr C Wahlbeck K Welbel M Bogic M Good practice in health care for migrants: views and experiences of care professionals in 16 European countries BMC Public Health 2011 11 187 10.1186/1471-2458-11-187 21439059 PMC3071322

[B20] Devillé W Greacen T Bogic M Dauvrin M Dias S Gaddini A Jensen NK Karamanidou C Kluge U Mertaniemi R Puigpinós i Riera R Sárváry A Soares JFF Stankunas M Straßmayr C Welbel M Priebe S Health care for immigrants in Europe: is there still consensus among country experts about principles of good practice? A Delphi study BMC Public Health 2011 11 699 10.1186/1471-2458-11-699 21914194 PMC3182934

[B21] Gold M Beyond Coverage and Supply: Measuring Access to Healthcare in Today's Market Health Serv Res 2008 33 3 625 652 PMC1975648 9685110

[B22] Bischoff A Bovier PA Rrustemi I Gariazzo F Eytan A Loutan L Language barriers between nurses and asylum seekers: their impact on symptom reporting and referral Soc Sci Med 2003 57 3 503 512 10.1016/S0277-9536(02)00376-3 12791492

[B23] Scheppers E van Dongen E Geertzen J Dekker J Potential barriers to the use of health services among ethnic minorities: a review Fam Pract 2006 23 3 325 348 10.1093/fampra/cmi113 16476700

[B24] Schoevers MA Loeffen MJ Van Den Muijsenbergh ME Lagro-Janssen ALM Health care utilisation and problems in accessing health care of female undocumented immigrants in the Netherlands Int J Public Health 2010 55 5 421 428 10.1007/s00038-010-0151-6 20502936 PMC2941053

[B25] van Doorslaer E Wagstaff A van der Burg H Christiansen T De Graeve D Duchesne I Gerdtham UG Gerfin M Geurts J Gross L Equity in the delivery of health care in Europe and the US J Health Econ 2000 19 553 583 10.1016/S0167-6296(00)00050-3 11184794

[B26] Grimm JW Wells JL Illegal Immigrants in the Emergency Department: An Ethical Dilemma for Nurses? J Emerg Nurs 2009 35 2 127 128 10.1016/j.jen.2008.08.018 19285176

[B27] Rousseau C ter Kuile S Munoz M Nadeau L Ouimet MJ Kirmayer L Crépeau F Health care access for refugees and immigrants with precarious status - Public health and human right challenges Can J Public Health 2008 99 4 290 292 18767273 10.1007/BF03403757 PMC6976187

[B28] Paez KA Allen JK Carson KA Cooper LA Provider and clinic cultural competence in a primary care setting Soc Sci Med 2008 66 5 1204 1216 10.1016/j.socscimed.2007.11.027 18164114 PMC2426909

[B29] Staton LJ Desbiens NA Physicians' knowledge of laws affecting medical decision making for seriously ill patients J Gen Intern Med 2007 22 Suppl 1 8 8

[B30] Walerius T Hill PD Anderson MA Nurses' Knowledge of Advance Directives, Patient Self-determination Act, and Illinois Advance Directive Law Clin Nurse Specialist 2009 23 6 316 320 10.1097/NUR.0b013e3181be3273 19858904

[B31] Hajjaj FM Salek MS Basra MKA Finlay AY Non-clinical influences on clinical decision-making: a major challenge to evidence-based practice J R Soc Med 2010 103 5 178 187 10.1258/jrsm.2010.100104 20436026 PMC2862069

[B32] Strech D Persad G Marckmann G Danis M Are physicians willing to ration health care? Conflicting findings in a systematic review of survey research Health Policy 2009 90 2-3 113 124 10.1016/j.healthpol.2008.10.013 19070396 PMC3635950

[B33] Washington D Bowles J Saha S Horowitz CR Moody-Ayers S Brown AF Stone VE Cooper LA Transforming Clinical Practice To Eliminate Racial-Ethnic Disparities in Healthcare J Gen Intern Med 2008 23 5 685 691 10.1007/s11606-007-0481-0 18196352 PMC2324135

[B34] Watt S Sword W Krueger P Implementation of a health care policy: An analysis of barriers and facilitators to practice change BMC Health Serv Res 2005 5 53 10.1186/1472-6963-5-53 16102173 PMC1201138

[B35] Weiner SJ Laporte M Abrams RI Moswin A Warnecke R Rationing access to care to the medically uninsured - The role of bureaucratic front-line discretion at large healthcare institutions Med Care 2004 42 4 306 312 10.1097/01.mlr.0000118706.49341.49 15076806

[B36] Hurst SA Slowther AM Forde R Pegoraro R Reiter-Theil S Perrier A Garrett-Mayer E Danis M Prevalence and determinants of physician bedside rationing: Data from Europe J Gen Inter Med 2006 21 11 1138 1143 10.1111/j.1525-1497.2006.00551.x PMC1831659 16836629

[B37] Harmsen JA Bernsen RM Bruijnzeels MA Meeuwesen L Patients' evaluation of quality of care in general practice: What are the cultural and linguistic barriers? Patient Educ Couns 2008 72 1 155 162 10.1016/j.pec.2008.03.018 18485657

[B38] Nierkens V Krumeich A de Ridder R van Dongen M The future of intercultural mediation in Belgium Patient Educ Couns 2002 46 4 253 259 10.1016/S0738-3991(01)00161-6 11932124

[B39] Kogan SM Wejnert C Chen YF Brody GH Slater LM Respondent-Driven Sampling With Hard-to-Reach Emerging Adults: An Introduction and Case Study With Rural African Americans J Adolesc Res 2011 26 1 30 60 10.1177/0743558410384734

[B40] Jensen NK Norredam M Draebel T Bogic M Priebe S Krasnik A Providing Medical Care for Undocumented Migrants in Denmark: What Are the Challenges for Health Professionals? BMC Health Serv Res 2011 11 154 10.1186/1472-6963-11-154 21711562 PMC3150245

[B41] Green J Thorogood N Qualitative Methods for Health Research 2004 First London: Sage

[B42] Hsieh HF Shannon SE Three approaches to qualitative content analysis Qual Health Res 2005 15 9 1277 1288 10.1177/1049732305276687 16204405

[B43] Miles MB Huberman AM Qualitative data analysis. An Expanded Sourcebook 1994 Thousand Oaks, CA: Sage

[B44] Patton MQ Qualitative research and evaluation methods 2002 Thousand Oaks, CA: Sage

[B45] Silverman D Interpreting qualitative data: Methods for analyzing talk, text, and interaction 2001 London: Sage

